# Regulation of the Membrane Trafficking of the Mechanosensitive Ion Channels TRPV1 and TRPV4 by Zonular Tension, Osmotic Stress and Activators in the Mouse Lens

**DOI:** 10.3390/ijms222312658

**Published:** 2021-11-23

**Authors:** Yosuke Nakazawa, Rosica S. Petrova, Yuki Sugiyama, Noriaki Nagai, Hiroomi Tamura, Paul J. Donaldson

**Affiliations:** 1 Faculty of Pharmacy, Keio University, 1-5-30 Shibako Minato-ku, Tokyo 105-8512, Japan; yuuki.1998.dragon@keio.jp (Y.S.); tamura-hr@a7.keio.jp (H.T.); 2 Department of Physiology, School of Medical Sciences, New Zealand Eye Centre, University of Auckland, 85 Park Road, Auckland 1023, New Zealand; r.petrova@auckland.ac.nz (R.S.P.); p.donaldson@auckland.ac.nz (P.J.D.); 3 Faculty of Pharmacy, Kindai University, 3-4-1 Kowakae, Higashi-Osaka, Osaka 577-8502, Japan; nagai_n@phar.kindai.ac.jp

**Keywords:** lens, zonular tension, osmotic stress, cell volume, mechanosensors, TRPV1/4

## Abstract

Lens water transport generates a hydrostatic pressure gradient that is regulated by a dual-feedback system that utilizes the mechanosensitive transient receptor potential vanilloid (TRPV) channels, TRPV1 and TRPV4, to sense changes in mechanical tension and extracellular osmolarity. Here, we investigate whether the modulation of TRPV1 or TRPV4 activity dynamically affects their membrane trafficking. Mouse lenses were incubated in either pilocarpine or tropicamide to alter zonular tension, exposed to osmotic stress, or the TRPV1 and TRPV4 activators capsaicin andGSK1016790A (GSK101), and the effect on the TRPV1 and TRPV4 membrane trafficking in peripheral fiber cells visualized using confocal microscopy. Decreases in zonular tension caused the removal of TRPV4 from the membrane of peripheral fiber cells. Hypotonic challenge had no effect on TRPV1, but increased the membrane localization of TRPV4. Hypertonic challenge caused the insertion of TRPV1 and the removal of TRPV4 from the membranes of peripheral fiber cells. Capsaicin caused an increase in TRPV4 membrane localization, but had no effect on TRPV1; while GSK101 decreased the membrane localization of TRPV4 and increased the membrane localization of TRPV1. These reciprocal changes in TRPV1/4 membrane localization are consistent with the channels acting as mechanosensitive transducers of a dual-feedback pathway that regulates lens water transport.

## 1. Introduction

The transparent and refractive properties of the ocular lens are established, respectively, by a cellular architecture that minimizes light scattering and a tissue geometry, which, in combination with the presence of a gradient of refractive index, sets the optical power of the lens [1,2]. However, the lens is not a purely passive optical element but rather a biological tissue that requires its steady-state lens transparency and refractive properties to be actively maintained by a unique cellular physiology [2,3,4]. Furthermore, primate lenses through the process of accommodation are able to dynamically change their optical power [5], thereby enabling the eye to focus on near and far objects. In recent years, several experiments have shown that lens water transports generates a substantial and highly regulated hydrostatic pressure gradient [6,7,8,9,10], which appears to be involved in the maintenance of steady-state lens transparency and power and may even play a role in the process of lens accommodation [11].

As a large avascular tissue, the lens has evolved an internal microcirculation system [2,3,4] which compensates for the absence of a blood supply by generating circulating fluxes of ions and water that deliver nutrients to and remove metabolic wastes from, the lens core faster than would occur by passive diffusion alone [12]. This microcirculation system is generated by a circulating flux of Na^+^ that drives an isotonic fluid flow that enters the lens at both the anterior and posterior poles via an extracellular pathway. Na^+^ and water then cross the membranes of deeper fiber cells before returning to the surface via an intracellular pathway mediated by the gap junction channels that direct Na^+^ and water to the lens equator, where Na^+^/K^+^ ATPase and aquaporin water channels are localized, to mediate their exit from the lens. This microcirculation has been shown to actively remove water from the lens [13], hence contributing to the maintenance of fiber cell volume (geometry), the water-to-protein ratio (refractive index), and, therefore, the overall optical properties of the lens [14].

**Figure 1:**
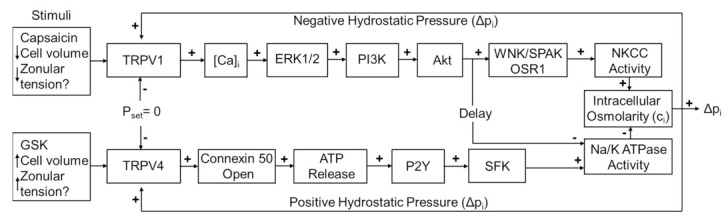
The dual-feedback control system that maintains hydrostatic pressure in the lens. Lens surface pressure (p_set_) is maintained by the competing activities of the two arms of a dual-feedback system that regulate ion transporters that control the intracellular osmolarity of cells at the lens surface. Increases in pressure (D_pi_), hypoosmotic stress, increased zonular tension, or the TRPV4 agonist GSK1016790A (GSK), all work via TRPV4 to activate a signaling pathway that involves the release of ATP via hemichannel, the subsequent activation of purinergic P2Y receptors, and the Src family of protein tyrosine kinases (SFK) to increase the activity of the Na^+^/K^-^ ATPase and decrease lens pressure. Decreases in pressure (D_pi_), hyperosmotic stress, decreased zonular tension or the TRPV1 agonist capsaicin all work via TRPV1 to activate the extracellular signal-regulated kinase 1/2 (ERK1/2), phosphatidylinositol 3-kinase (PI3K/Akt) and the WNK (Kinase with no lysine (K)), and SPAK (Ste20-related proline-alanine-rich kinase)/OSR1(oxidative stress-responsive kinase-1) signaling pathway to directly activate the sodium potassium dichloride cotransporter (NKCC) and to eventually reduce the decrease in the activity of the Na^+^/K^-^ ATPase to effect an increase in surface pressure. This scheme is based on earlier model [7,18].

An additional consequence of the microcirculation system is that the outflow of water through gap junction generates an internal hydrostatic pressure gradient [6], which is regulated by a dual-feedback system that utilizes the transient receptor potential vanilloid (TRPV) channels, TRPV1 and TRPV4 [7]. In this system, TRPV1 and TRPV4 reciprocally transduce changes in lens pressure into the modulation of ion transporter activity in order to effect alterations in circulating ion and water fluxes that act to restore the pressure gradient (Figure 1). An increase in lens pressure activates TRPV4 channels, which in turn causes an increase in Na^+^/K^+^-ATPase activity [7], while a decrease in pressure activates a TRPV1-mediated increase in the activity of the sodium potassium dichloride cotransporter (NKCC) and a secondary delayed decrease in Na^+^/K^+^-ATPase activity [15]. In this system, the direct pharmacological activation of one of the arms of the system by either capsaicin (TRPV1) or GSK101670A (GSK101: TRPV4) was shown to induce an initial change in pressure that subsequently returned to baseline due the reciprocal pressure-sensitive activation of the other arm of the feedback pathway (Figure 1). This biphasic response suggests that the TRPV1- and TRPV4-mediated arms of the dual-feedback system exhibit strong crosstalk.

In this regard, it is interesting that the TRPV1- and TRPV4-mediated signaling pathways activated by changes in lens pressure are also activated in response to osmotic challenge. Exposing lenses to hypoosmotic challenge has been shown to activate TRPV4 and increase Na^+^/K^+^-ATPase activity through an Src family tyrosine kinase-dependent mechanism [16,17], while culturing lens in hyperosmotic solutions caused a TRPV1-dependent increase in NKCC activity that was mediated by a phosphoinositide 3-kinase (PI3K) signaling pathway [18]. Similar reciprocal roles for TRPV1 and TRPV4 in the sensing of changes in cell volume and effecting changes to ion transport to restore cell volume have been observed in a variety of other cell types [19,20]. In the lens, this regulatory system would appear to be critical to the ability of the lens to maintain the cellular order that underpins its transparent properties [21].

Recently, in addition to osmotic challenge, we have also shown that altering the tension applied to the lens can also alter the lens pressure gradient [11]. In the eye, the lens is suspended in place by the zonules of Zinn that connect the lens to the muscles of the ciliary body. In primates and humans, the contraction of the ciliary muscle and the resultant changes in the tension applied to the lens induce the changes in lens geometry that drive the process of accommodate [5]. In rodents, we have shown that the pharmacological modulators of ciliary muscle contractility, pilocarpine and tropicamide can decrease and increase, respectively, the circumlental space between the ciliary process and the lens to alter the resting tension applied to the lens [11,22]. The pilocarpine-induced reduction in zonular tension caused an increase in lens pressure, which was abolished by the inhibition of TRPV1, while the increase in zonular tension induced by tropicamide resulted in a decrease in lens pressure that was mediated by TRPV4 [11]. Interestingly, rather than producing the biphasic change in lens pressure observed following either the pharmacological or osmotic activation of TRPV1 and TRPV4, altering the zonular tension produced a sustained shift in the pressure gradient. Taken together, it is evident that a variety of stimuli utilize TRPV1 and TRPV4 channels to not only activate signaling pathways to maintain a relative constant lens pressure gradient, but also to alter the steady-state magnitude of the gradient.

Because of the emerging importance of TRPV1 and TRPV4 channels to overall lens function, we conducted a comprehensive mapping of the expression of the two channels in the mouse lens [23]. This study revealed that while both TRPV1 and TRPV4 proteins are expressed throughout the whole lens, their subcellular distribution changed as a function of fiber cell differentiation. In peripheral fiber cells in the lens outer cortex, TRPV1 and TRPV4 labelling was predominately associated with the cytoplasm, while in the deeper regions of the lens, TRPV1 and TRPV4 labelling was found to be associated with the fiber cell membranes. However, in peripheral fiber cells, the subcellular location of TRPV4, but not TRPV1, could be dynamically shifted from the cytoplasm to the membrane, if the lenses were first fixed in situ in the eye with their zonules attached [23]. These results suggested that the change in the subcellular localization of TRPV4, and hence functionality of TRPV4, could be regulated by changes in zonular tension, which can in turn can be transduced into changes in lens pressure. Since in addition to changes in zonular tension, direct pharmacological activation and osmotic challenge can also alter lens pressure via TRPV1 and TRPV4 channels, in this current paper, we investigated whether these other stimuli can also alter the membrane trafficking of TRPV1 and TRPV4 in peripheral fiber cells. Our results show that the different stimuli had differential and often reciprocal effects on the trafficking of TRPV1 and TRPV4 to and from the membrane. These observed changes in TRPV1 and TRPV4 membrane localization, and presumably functionality, are consistent with the two channels acting as mechanosensitive transducers of dual-feedback signaling pathways that exhibit a degree of pathway crosstalk to regulate water transport in the mouse lens.

## 2. Results

**Figure 2:**
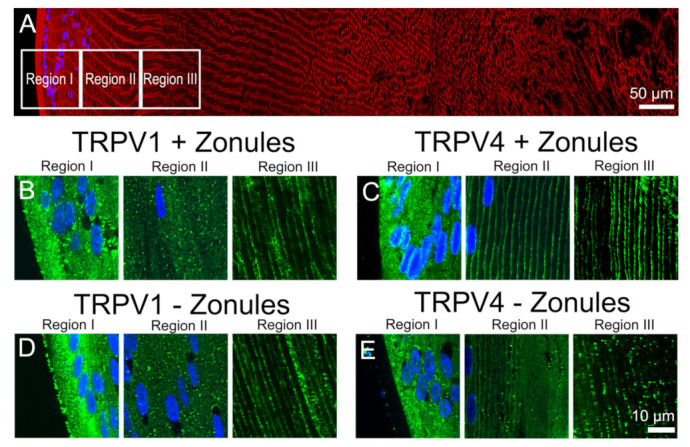
Effect of cutting lens zonules on the subcellular localization of TRPV1 and TRPV4 labelling in the outer cortex of the mouse lens. (**A**). Overview montage of the mouse lens sectioned axially and labelled with the membrane marker wheatgerm agglutin (WGA, red) and nuclei marker 4,6-Diamidino-2-phenylindole (DAPI, blue), showing the three regions (white boxes) in the outer cortex from where the high-magnification images shown in (**B**–**E**) were captured. (**B**–**E**) High-power images from the three regions defined in panel (**A**) obtained from lenses organ-cultured with (**B**,**C**) or without (**D**,**E**) their zonules attached, which were labelled with DAPI and either TRPV1 (**B**,**D**) or TRPV4 (**C**,**E**) antibodies (green).

In a previous study, we mapped the subcellular distribution of TRPV1 and TRPV4 throughout all regions of the lens and showed that both channels are predominately cytoplasmic in the peripheral fiber cells in the outer cortex but reside in the plasma membranes of deeper fiber cells in the inner cortex and nucleus of the mouse lens [23]. In this study, we also showed that the subcellular distribution of TRPV4, but not TRPV1, can be altered in a localized region of the outer cortex by mechanically cutting the zonules [23]. In this current study, we focus on this localized peripheral region in outer cortex of the mouse lens (Figure 2A) and show that alterations in membrane localization of both TRPV1 and TRPV4 in this localized region of the mouse lens can be dynamically and differentially regulated by a variety of stimuli.

### 2.1. Alteration of the Lens Tension Dynamically Changes the Membrane Localization of Trpv4 but Not Trpv1

**Figure 3:**
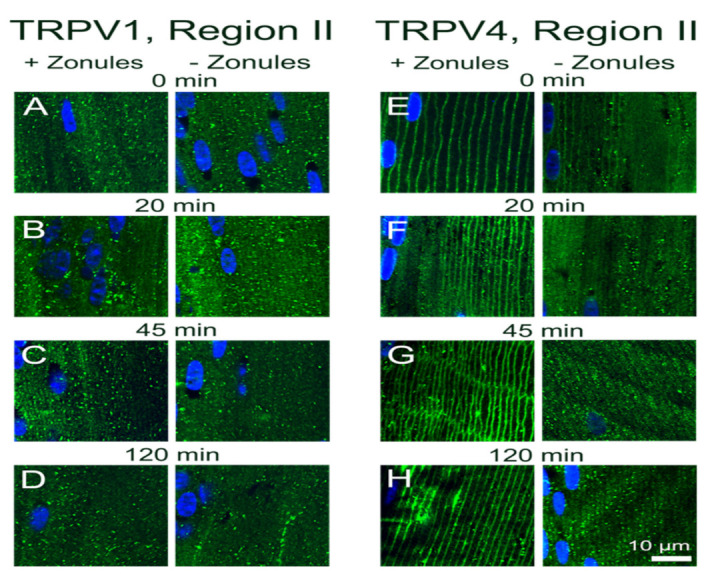
Time course showing the effect of cutting lens zonules on the subcellular localization of TRPV1 and TRPV4 labelling in the outer cortex mouse lens. Images captured from axial sections obtained from region II of the outer cortex of mouse lenses organ-cultured with or without their zonules attached for 3 min (**A**,**E**), 20 min (**B**,**F**), 45 min (**C**,**G**) or 120 min (**D**,**H**). (**A**–**D**) TRPV1 labelling (green) in region II is cytoplasmic and unaffected by changes in zonular tension. (**E**–**H**) TRPV4 labelling (green) in region II is membranous (left) in lenses cultured in the presence of zonular tension but is removed from the membrane over time in lenses organ-cultured after cutting the zonules. Nuclei are labelled with DAPI (blue).

In Figure 2, we present confirmation of the previous observations of Nakazawa et al. 2019 [23]. In mouse lenses organ-cultured for 120 min under in situ conditions that maintain zonular tension, TRPV1 receptors were localized in the cytoplasm (Figure 2B), while TRPV4 receptors were localized in the membrane (Figure 2C) of the newly differentiated fiber cells in region II of the outer cortex. However, if lenses were organ-cultured after first having their zonules cut to remove the lens from the eye, TRPV channel-specific changes in the subcellular labelling pattern were observed. While cutting the zonules had no effect on the labelling pattern observed for TRPV1 in all three regions of the outer cortex (Figure 2B,D), cutting the zonules to release the tension applied to the lens changed the labelling for TRPV4 observed in region II changed from membranous to cytoplasmic (Figure 2C,E). To further study this trafficking of TRPV4 to the membrane in response to the mechanical release of zonular tension, a time course of the changes in the subcellular distribution of TRPV1 and TRPV4 labelling in region II in the presence and absence of zonular tension was collected (Figure 3). These time course experiments confirmed that the state of zonular tension had no effect on the primarily cytoplasmic localization of TRPV1 in region II of the outer cortex of the mouse lens (Figure 3A–D). By contrast, TRPV4 labelling in region II obtained from lenses incubated in situ to maintain zonular tension remained membranous over the whole 120 min period in organ culture (Figure 3E–H, left panels). However, upon cutting the zonules and culturing the lens in the absence of applied zonular tension, TRPV4 rapidly became cytoplasmic in region II within 20 min of cutting the zonules (Figure 3E–H, right panels). In fact, signs of removal of TRPV4 from the membrane were evident at the zero time point (Figure 3E), presumably due to the time delay (estimated at around 3 min) associated with removal of the lens from the eye, and its first immersion in fixative. These experiments show that when maintained in situ, TRPV4 is located in the membranes of peripheral fiber cells located in region II of the outer cortex and that mechanically cutting the lens zonules induces a very rapid removal of TRPV4, but not TRPV1, from the membranes of cells specifically located in this region of the outer cortex of the mouse lens.

**Figure 4:**
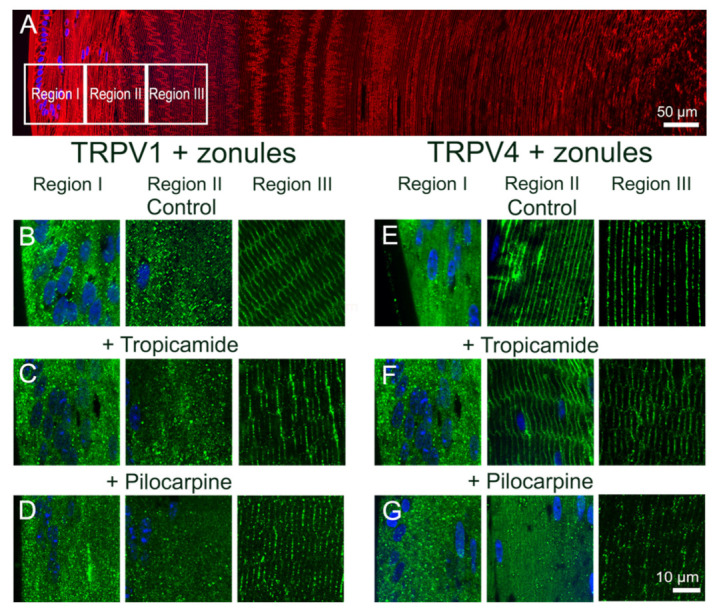
Effect of pharmacological modulation of zonular tension on the subcellular localization of TRPV1 and TRPV4 labelling in the outer cortex of the mouse lens. (**A**). Overview montage of the mouse lens sectioned axially and labelled with the membrane marker WGA (red) and nuclei marker DAPI (blue), showing the three regions (white boxes) in the outer cortex from where the high-magnification images shown in (**B**–**G**) were captured. (**B**–**G**) High-power images from the three regions defined in panel (**A**) obtained from lenses organ-cultured in situ to maintain zonular tension in the absence (**B**,**E**) or presence tropicamide (**C**,**F**) or pilocarpine (**D**,**G**) showing TRPV1 (**B**–**D**) and TRPV4 (**E**–**G**) labelling (green). Nuclei are labelled with DAPI (blue).

**Figure 5:**
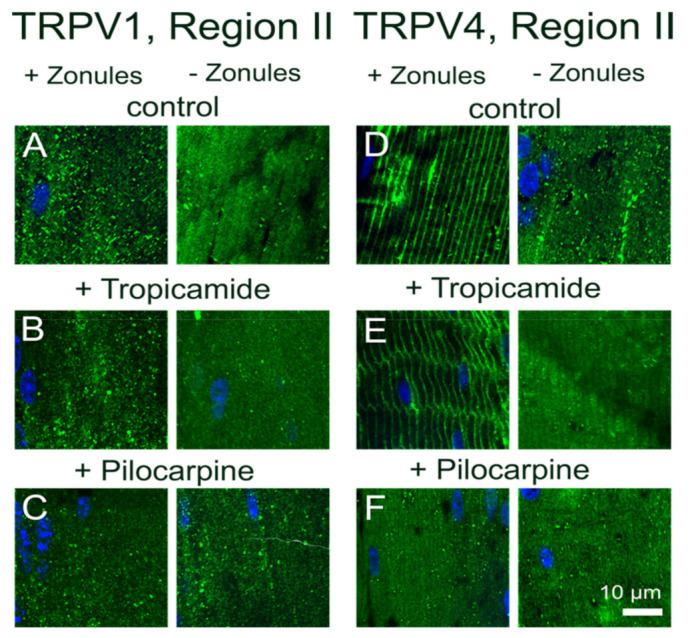
Effect of pharmacological modulation of zonular tension on the subcellular localization of TRPV1 and TRPV4 labelling in the outer cortex of the mouse lenses organ-cultured with and without zonules. Images captured from axial sections obtained from region II of the outer cortex of mouse lenses organ-cultured either in situ (+Zonules) or ex vitro (−Zonules) in the absence (**A**,**D**) or presence tropicamide (**B**,**E**) or pilocarpine (**C**,**F**). (**A**–**C**) The addition of tropicamide (**B**) or pilocarpine (**C**) in the presence (left panels) or absence (right panels) zonular tension had no effect on the cytoplasmic labelling of TRPV1 (green) in region II of the outer cortex. (**D**–**F**) The addition of tropicamide (**E**) in the presence (left panels) or absence (right panels) of zonular tension had no effect on the subcellular distribution of TRPV4 (green). However, pilocarpine addition to lenses organ-cultured with their zonules intact ((**F**), left panel) induced a shift of TRPV4 labelling to the cytoplasm. Nuclei are labelled with DAPI (blue).

To further examine whether this localized change in the membrane localization of TRPV4 in region II of the outer cortex caused by the cutting the lens zonules was indeed due to a change in the tension applied to the lens and not due to nonspecific mechanical damage, we adopted a pharmacological approach to modulate zonular tension. This was achieved by organ-culturing lenses in situ in the absence or presence of either tropicamide or pilocarpine, to modulate the contractility of the ciliary muscle to increase or decrease, respectively, the circumlental space between the lens and ciliary processes, and as a result the tension applied to the lens [11,22]. Using this approach to either increase (tropicamide) or decrease (pilocarpine) the tension applied to the lens had no effect on the subcellular distribution of TRPV1, which remained cytoplasmic in fiber cells localized to region II of the outer cortex (Figure 4C,D). Increasing the zonular tension applied to the lens using tropicamide also had no effect on the subcellular distribution of TRPV4 in region II, which remained membranous in lenses with their zonules attached (Figure 4F). However, culturing lenses in situ in the presence of pilocarpine to reduce zonular tension induced a shift in TRPV4 labelling from the membrane to the cytoplasm (Figure 4G). As might be expected, tropicamide and pilocarpine had no effect on the subcellular distribution of TRPV4 in lenses organ-cultured in vitro, which had been separated from the ciliary body to facilitate their removal from the eye (Figure 5). This comparison reinforces that the removal of TRPV4 from the membrane occurs via the action of pilocarpine on the ciliary muscle to reduce the zonular tension applied to the lens (Figure 5F, left panel) and not via a direct action of pilocarpine on the lens itself via muscarinic receptors known to be expressed in the lens [24].

Collectively, these experiments show that alterations to the tension applied to the lens, induced by either mechanically cutting the zonules or pharmacologically modulating ciliary muscle contractility, can dynamically modulate the membrane trafficking of TRPV4, but not TRPV1, in region II of outer cortex of the mouse lens.

### 2.2. Osmotic Stress Induces Differential Changes in the Membrane Localization of TRPV1 and TRPV4

**Figure 6:**
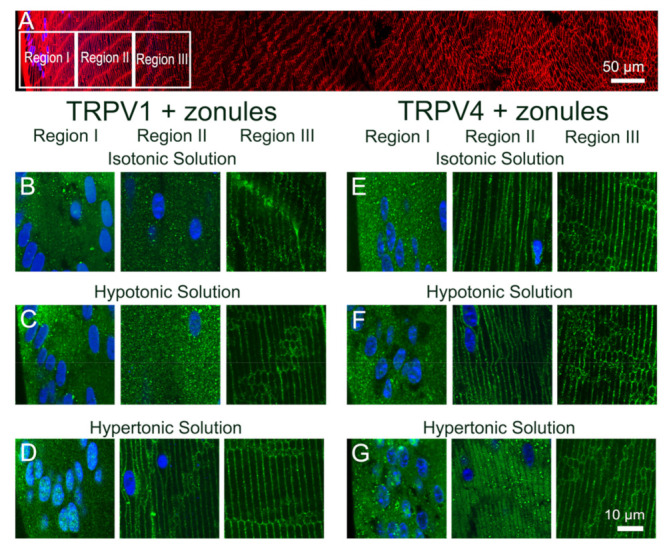
Effect of osmotic challenge on the subcellular localization of TRPV1 and TRPV4 labelling in the outer cortex of the mouse lens. (**A**). Overview montage of the mouse lens sectioned axially and labelled with the membrane marker WGA (red) and nuclei marker DAPI (blue), showing the three regions (white boxes) in the outer cortex from where the high-magnification images shown in (**B**–**G**) were captured. (**B**–**G**) High-power images from the three regions defined in panel A obtained from lenses organ-cultured in situ to maintain zonular tension in the presence of isotonic (**B**,**E**), hypotonic (**C**,**F**) or hypertonic (**D**,**G**), AAH showing TRPV1 (**B**–**D**) and TRPV4 (**E**–**G**) labelling (green). Nuclei are labelled with DAPI (blue).

**Figure 7:**
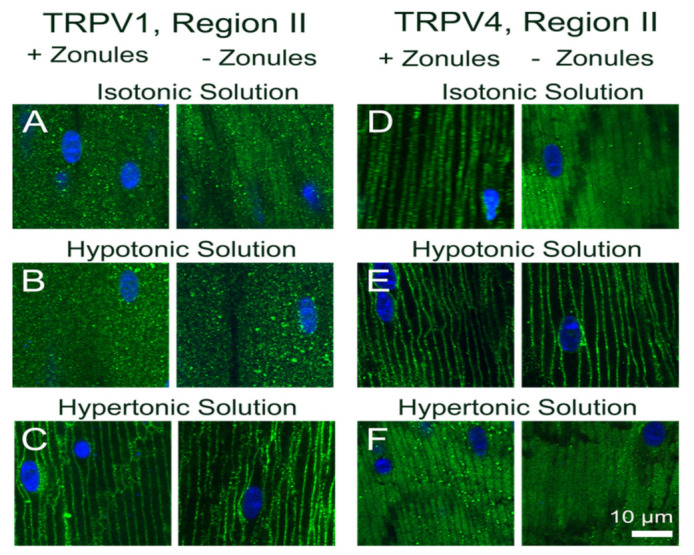
Effect of osmotic challenge on the subcellular localization of TRPV1 and TRPV4 labelling in the outer cortex of the mouse lenses organ-cultured with and without zonules. Images captured from axial sections obtained from region II of the outer cortex of mouse lenses organ-cultured either in situ (+Zonules) or ex vitro (−Zonules) in the presence of isotonic (**A**,**D**), hypotonic (**B**,**E**) or hypertonic (**C**,**F**) AAH. (**A**–**C**) TRPV1 was found cytoplasmic in lenses incubated in isotonic (**A**) and hypotonic (**B**) AAH, while it was found membranous in lenses treated with hypertonic (**C**) AAH regardless of the presence (left panels) or absence (right panels) of zonular tension. (**D**–**F**) TRPV4 membrane labelling in lenses organ-cultured in situ with (left panels) or without (right panels) zonules attached was unchanged by hypotonic challenge (**E**); however, a shift of TRPV4 labelling to the cytoplasm was observed following hypertonic exposure (**F**, left panel), which was not dependent on the presence of zonular tension (**F**, right panel). Nuclei are labelled with DAPI (blue).

To investigate whether osmotic stress effects the subcellular localization of TRPV1 and TRPV4 channels, lenses were organ-cultured in situ and exposed to either a hypoosmotic or hyperosmotic challenge for 120 min to induce lens swelling and shrinkage, respectively. We found that in lenses organ-cultured in situ to maintain zonular tension, osmotic challenge had no effect on the subcellular distribution of TRPV1 and TRPV4 in regions I and III of the outer cortex but induced differential changes in membrane localization of both TRPV1 and TRPV4 in region II (Figure 6). These changes in region II are highlighted in Figure 7 and compared to the changes in TRPV1 and TRPV4 membrane localization induced by exposing lenses organ-cultured with their zonules cut to osmotic challenge. The cytoplasmic labelling observed for TRPV1 in lenses cultured under isosmotic conditions, with or without their zonules attached (Figure 7A), was unchanged by exposure to hypoosmotic challenge solution (Figure 7B). However, the exposure to hyperosmotic challenge induced a trafficking of TRPV1 to the membranes of fiber cells in region II of the outer cortex that occurred in the presence and absence of zonular tension (Figure 7C). As seen above, the membrane localization of TRPV4 depended on the presence of zonular tension (Figure 7D, left panel), and this membrane localization was maintained upon hypotonic challenge (Figure 7E, left panel). However, in lenses with their zonules cut, the initial removal of TRPV4 from the membrane observed under isotonic conditions (Figure 7D, right panel) was reversed by hypotonic challenge to yield strong membrane labelling (Figure 7E, right panel), despite the absence of applied zonular tension. Finally, exposure of lenses to hypertonic challenge caused TRPV4 membrane labelling in region II of lenses with their zonules attached to adopt a more cytoplasmic labelling pattern (Figure 7F, left panel) but had no effect on the cytoplasmic labelling already observed for TRPV4 in lens that had had their zonules cut (Figure 7F, right panel).

Taken together these experiments show that in addition to the presence of zonular tension, the membrane localization of TRPV4 can be increased by hypotonic lens swelling, while its membrane localization in region II was reduced by decreases in zonular tension and hypertonic lens shrinkage. By contrast, the membrane localization of TRPV1 in region II seems to be insensitive to zonular tension but is increased by exposure to hypertonic lens shrinkage. These reciprocal changes in TRPV1 and TRPV4 membrane localization, and hence potentially functionality, support the notion that the two channels are part of a dual-feedback pathway that regulates both lens volume [17,18] and the internal hydrostatic pressure gradient recently measured in the mouse lens [7].

### 2.3. Pharmacological Activators of TRPV1/4 Induce Reciprocal Changes in the Membrane Localization of TRPV1 and TRPV4

**Figure 8:**
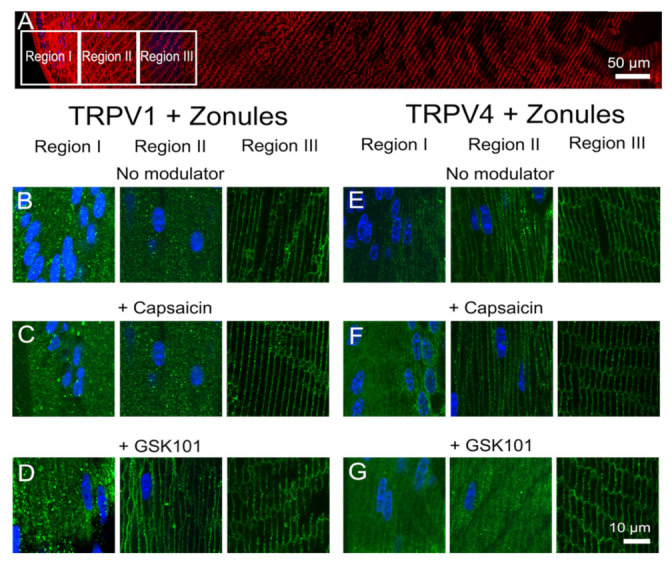
Effects of pharmacological activators of TRPV1 and TRPV4 activity on the subcellular localization of TRPV1 and TRPV4 in the outer cortex of the mouse lens. (**A**) Overview montage of the mouse lens sectioned axially and labelled with the membrane marker WGA (red) and nuclei marker DAPI (blue), showing the three regions (white boxes) in the outer cortex from where the high-magnification images shown in (**B**–**G**) were captured. (**B**–**G**) High-power images from the three regions defined in panel (**A**) obtained from lenses organ-cultured in situ to maintain zonular tension in the absence ((**B**,**E**) no modulator) or presence of either the TRPV1-activator capsaicin (**C**,**F**) or the TRPV4-activator GSK (**D**,**G**) showing TRPV1 (**B**–**D**) and TRPV4 (**E**–**G**) labelling (green). Nuclei are labelled with DAPI (blue).

**Figure 9:**
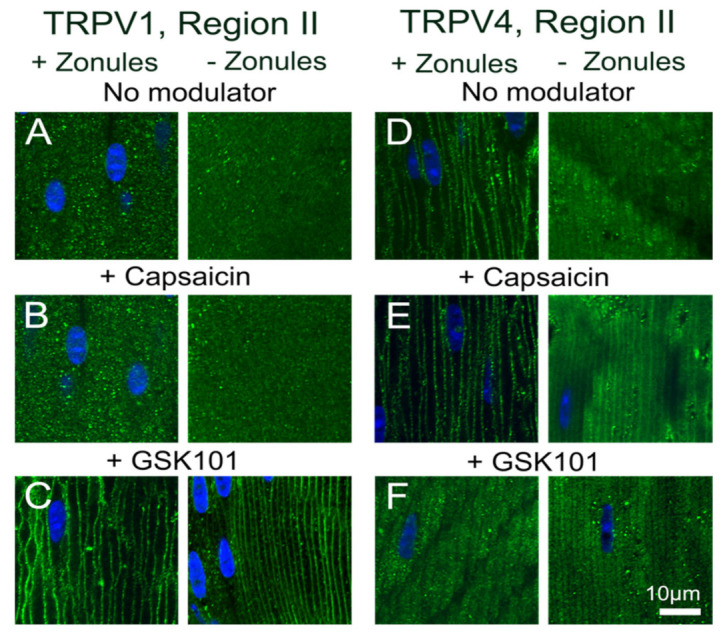
Effects of pharmacological activators of TRPV1 and TRPV4 activity on the subcellular localization of TRPV1 and TRPV4 in the outer cortex of the mouse lens organ-cultured with and without zonules. Images captured from axial sections obtained from region II of the outer cortex of mouse lenses organ-cultured either in situ (+Zonules) or ex vitro (−Zonules) in absence ((**A**,**D**) no modulator) or presence of either the TRPV1-activator capsaicin (**B**,**E**) or the TRPV4-activator GSK (**C**,**F**). (**A**–**C**) TRPV1 labelling is cytoplasmic in lenses treated with capsaicin (**B**) but exposure to GSK induces a shift in TRPV1 labelling to the membrane in lenses organ-cultured with and without zonules attached. (**D**–**F**) TRPV4 labelling in lenses cultured in situ with their zonules attached remained membranous following exposure to capsaicin (**E**, left panel), but TRPV4 labelling shifted to the cytoplasm by exposure to GSK (**F**, left panel), and these changes were unaffected by cutting the zonules (**E**,**F**, right panels). Nuclei are labelled with DAPI (blue).

To investigate potential crosstalk between the dual arms of the TRPV1- and TRPV4-mediated signaling pathways that regulate lens volume and hydrostatic pressure, we organ-cultured lenses in the absence and presence of pharmacological activators of either TRPV1 or TRPV4 channels and looked for changes in the subcellular distribution of the two channels in the outer cortex of the lens. In organ-cultured lenses in situ, to maintain zonular tension, the addition of the TRPV1-activator capsaicin, or the TRPV4-activator GSK101, had no effect on the subcellular distributions of TRPV1 or TRPV4 in regions I or III of the outer cortex (Figure 8), but once again differential changes were observed for the two channels in region II. While capsaicin had no effect on the membrane distribution of TRPV1 (Figure 8C), GSK101 caused a shift of TRPV1 from a cytoplasmic to a membranous labelling pattern (Figure 8D). While capsaicin maintained the membrane labeling for TRPV4 (Figure 8F), the addition of GSK101 induced a reduction in TRPV4 membrane labelling (Figure 8G). To assess whether the presence of zonular tension changes the effects of capsaicin or GSK101 on TRPV1/4 membrane localization, the labelling patterns in region II obtained from lenses with and without zonules were compared (Figure 9). While the presence or absence of zonular tension did not alter the inability of capsaicin to recruit TRPV1 from its cytoplasmic location to the membrane (Figure 9B), it did increase the membrane localization of TRPV4 in lenses with cut zonules (Figure 9E, right panel). By contrast, GSK101 induced a shift to the membrane for TRPV1 that was independent on the presence or absence of lens zonules (Figure 9C). While GSK101 removed TRPV4 from the membranes of fiber cells incubated in the activator with their zonules attached (Figure 9F, left panel), GSK101 had no effect on the cytoplasmic location of TRPV4 seen in lenses with their zonules cut. Thus, it appears that the pharmacological activation of TRPV1 can increase the membrane localization of TRPV4, while conversely, the activation of TRPV4 reduces its membrane localization and increases the membrane localization of TRPV1.

## 3. Discussion

Previous electrophysiological studies have established that the transport of water through the lens generates a substantial hydrostatic pressure gradient [6]. This pressure gradient was subsequently shown to be regulated by a dual-feedback pathway [7], which utilizes the mechanosensitive channels TRPV1 and TRPV4 to sense changes in cell volume [16,17,18] and zonular tension [11] (Figure 1), to effect changes in ion transport to alter lens pressure/water transport [7,15]. In this study, we utilized an immunohistochemistry approach to show that the membrane localization of TRPV1 and TRPV4, and presumably their functional activity, is dynamically regulated in a localized region in the outer cortex of the mouse lens. We found that reducing the tension applied to the lens by either cutting the zonules (Figure 2 and Figure 3), or pharmacologically modulating the contractility of the ciliary muscle with pilocarpine, caused TRPV4, but not TRPV1, to be removed from the membrane (Figure 4 and Figure 5). While exposure of lenses to osmotic challenge had reciprocal effects on the membrane localization of both TRPV1 and TRPV4 (Figure 6 and Figure 7), with hypotonic challenge causing an increase insertion of TRPV4 into fiber cell membranes. By contrast, exposure to hypertonic solutions initiated a removal of TRPV4 from the membrane and an insertion of TRPV1. Similar reciprocal effects on the membrane location of the two channels were observed following the application of specific pharmacological activators of TRPV1 and TRPV4 (Figure 8 and Figure 9). The application of the TRPV4-activator GSK101 induced the insertion of TRPV1 into the membrane, while in lenses with their zonules cut, the TRPV1 activator capsaicin induced the insertion of TRPV4 into the membrane. Taken together, these results suggest that the dynamic membrane trafficking of TRPV1 and TRPV4 mechanoreceptors plays a role in the regulation of lens pressure and therefore water transport in the lens.

Our results in the lens are broadly consistent with observations from a number of different tissues which have shown that in response to a wide variety of stimuli membrane vesicles containing TRPV1 or TRPV4 channels traffic to and from the plasma membrane in order to modulate the activity of the two channels [25]. These studies have shown that this trafficking of vesicles to the plasma membrane is a very rapid and highly regulated process that uses a cascade of protein interactions to ensure efficient delivery of TRPV1 and TRPV4 to the plasma membrane [26,27]. This exocytotic delivery of channels is counterbalanced by endocytic removal to ensure a constant turnover of the TRPV1 and TRPV4 to regulate their functionality [28]. It has been shown for TRPV4 channels that an initial rapid membrane insertion of membrane-associated TRPV4 vesicles occurs within a few seconds of activation of TRPV4 with GSK101 [29]. This initial exocytosis of TRPV4 was followed by a secondary endocytosis and accumulation of TRPV4 in recycling endosomes in the continued presence of GSK101 [30,31]. Similar removal from the membranes and rapid endocytosis is observed for TRPV1 channel after prolonged exposure to capsaicin [32]. In our experiments, which observed the distribution of TRPV1/4 channel in the lens after a 45 min incubation period, we have not captured these initial rapid events associated with the activation of TRPV4 by GSK101 but have observed the slower process of the secondary removal of TRPV4 from the membrane (Figure 9F). Furthermore, due to their longer duration, our experiments have revealed that a mechanism exists in the lens to reciprocally regulate the abundance and presumably the functionality of TRPV1 and TRPV4 channels in the membranes of fiber cells in the lens periphery (Figure 8 and Figure 9).

The observed reciprocal pharmacological regulation of TRPV1 and TRPV4 recruitment to the plasma membrane is consistent with the observed transient biphasic increase and decrease in surface hydrostatic pressure observed following the addition of capsaicin and GSK101, respectively [7,11]. These pressure measurements revealed that in the lens, TRPV1 and TRPV4 regulate the opposing arms of a dual-feedback system designed to maintain a constant hydrostatic pressure gradient in the lens [7]. Our current results tend to suggest that changes in the membrane trafficking of TRPV1 and TRPV4 are an inherent feature of how this dual-feedback system alters lens transport to maintain a constant lens pressure gradient. While our studies to date have not investigated the cellular signaling pathways responsible for this crosstalk between TRPV1 and TRPV4 membrane trafficking, we have shown that osmotic challenge and changes in zonular tension can also differentially affect the membrane trafficking of the two channels.

In a variety of tissues, the changes in membrane trafficking of both TRPV1 and TRPV4 have been implicated in the maintenance of steady-state cell volume [33]. TRPV4 insertion in the membrane has been shown to be increased in salivary glands [33] and astrocytes in the retina [34] in response to hypoosmotic stress in response to hypotonic challenge. By contrast, hyperosmotic stress has been shown to increase TRPV1 membrane insertion in retinal ganglion cells [35]. There is also strong evidence for the reciprocal activity of TRPV1 and TRPV4 in maintenance of lens volume. The exposure of porcine lenses to hypoosmotic challenge to induce lens swelling has been shown to activate a TRPV4-mediated signaling pathway that increases Na^+^/K^+^-ATPase activity and produces a regulatory volume decrease that restores lens volume [17]. By contrast, shrinking the lens by hyperosmotic challenge activates a TRPV1-mediated signaling cascade that induces sodium potassium dichloride cotransporter 1 (NKCC1) phosphorylation, which increases the uptake of Na^+^, K^+^ and Cl^−^ ions which drives a regulatory volume increase to restore lens volume [15]. In the current study, we showed that not only does hypotonic and hypertonic challenge increase the membrane insertion of TRPV4 and TRPV1, respectively, but hypertonicity also promotes the removal of TRPV4 from the membrane (Figure 7F). This reciprocal regulation of TRPV1 (insertion) and TRPV4 (removal) membrane trafficking by hypertonic challenge serves to reinforce the link between the two arms of the dual-feedback system that controls lens hydrostatic pressure. Furthermore, our observations support the contention that the overall transport of water (pressure) through the lens and the control of lens volume are reciprocally regulated by the same TRPV1- and TRPV4-mediated signaling pathways.

Previously, we showed that pharmacologically manipulating ciliary muscle contractility with either pilocarpine or tropicamide caused a sustained increase and decrease in the hydrostatic pressure gradient, respectively [11]. The pilocarpine-mediated decrease in zonular tension was shown to be mediated by TRPV1 but lacked the biphasic response observed when TRPV1 was directly activated by capsaicin. Although pilocarpine did not noticeably change the membrane localization of TRPV1, which was likely due to the rapid activation of TRPV1 followed by its subsequent removal from the membrane during the 2 h incubation period, it did alter the membrane location of TRPV4 (Figure 4 and Figure 5). This suggests that the observed pilocarpine-induced sustained increase in pressure was caused by the removal of TRPV4 from the membrane that effectively blocked the activation of the TRPV4-mediated arm of the dual-feedback pathway, which would normally counter the TRPV1-induced pressure increase. By contrast, the tropicamide-mediated increase in zonular tension induced a sustained decrease in the hydrostatic pressure gradient that was shown to be mediated by TRPV4 [11]. Here, unlike the direct pharmacological stimulation of TRPV4 with GSK101 (Figure 8G and Figure 9F), the change in membrane tension induced by tropicamide caused TRPV4 to not only be retained in the membrane but inhibited the GSK101-mediated recruitment of TRPV1 to the membrane (Figure 4 and Figure 5). This inhibition of TRPV1 membrane insertion appeared to block the TRPV1-mediated arm of the dual-feedback pathway, which normally counteracts the TRPV4-induced decrease in pressure, to produce the observed sustained decrease in lens pressure gradient [11].

This ability of lens pressure to respond to changes in zonular tension induced by muscarinic modulation of the ciliary muscle implies that the transport of water through the mouse lens driven by the microcirculation system can be dynamically regulated by stimuli external to the lens. In this regard, it is well known that the processes of accommodation can rapidly change the shape of and overall optical power of primate lenses [36], but the results from our current and previous studies suggest that changes in zonular tension may also have effects on water transport and therefore the optical properties of the non-accommodating mouse lens. Not only have changes in zonular tension induced by pilocarpine been shown to remove TRPV4 from the membrane (Figure 4 and Figure 5) and increase the hydrostatic pressure gradient [11], they have also been shown to induce the removal of the water channel aquaporin 5 (AQP5) from the membranes of peripheral fiber cells located in the anterior influx pathway and equatorial efflux zones, but not in the posterior influx pathway [22]. Since in other tissues, TRPV1/4 activation has been linked to AQP5 membrane trafficking that increases water permeability [33] and in the lens (unpublished study from our group), we would speculate that changes in zonular tension would induce localized changes in water permeability that alter the relative contributions of water influx via the anterior and posterior poles, which would in turn change the curvature of the anterior surface of the aspheric mouse lens. Such changes in lens geometry would in turn alter the optical power of the lens and its ability to focus onto the retina. Hence, we envisage that the ability of changes in zonular tension to shift the steady-state hydrostatic pressure gradient is part of a mechanism that ensures light remains focused on the retina as the lens and/or eye grows. If correct, this implies that the lens is actively involved in the process of emmetropisation and dysfunction of this process can lead to the onset of myopia.

## 4. Materials and Methods

### 4.1. Materials

Rabbit anti-TRPV1 C-terminus antibody (amino acid residues 824–838 of rat TRPV1 protein) was purchased from Alomone Labs (#ACC-030, Jerusalem, Israel). Rabbit anti-TRPV4 C-terminus antibody (amino acid residues 880 to the C-terminus of mouse TRPV4 protein) was obtained from Abcam (#ab39260, Cambridge, MA, USA). Goat antirabbit secondary antibody conjugated to Alexa Fluor 488 and the membrane marker Wheat Germ Agglutinin (WGA) conjugated to Alexa Fluor 594 were both purchased from Thermo Fisher Scientific (Waltham, MA, USA). 4,6-Diamidino-2-phenylindole (DAPI), used to stain the nucleus, was obtained from Dojindo (Kumamoto, Japan). Tropicamide (1% *w*/*v*), or pilocarpine (1% *w*/*v*) obtained from Sigma-Aldrich (St. Louis, MO, USA) were used to relax or contract the ciliary muscles, respectively [37]. Capsaicin, a TRPV1 agonist, and GSK1016790A (GSK101), a TRPV4 agonist, were purchased from Sigma-Aldrich (St. Louis, MO, USA). Paraformaldehyde, NaCl, MgCl_2_, NaHCO_3_, CaCl_2_, glucose, sucrose, HEPES and D-mannitol were purchased from Nakalai Tesque (Kyoto, Japan). PBS (130 mM NaCl, 3 mM KCl, 10 mM Na_2_HPO_4_, 2 mM KH_2_PO_4_, pH 7.4) was made from tablets Sigma-Aldrich (St. Louis, MO, USA). Isotonic artificial aqueous humor (AAH) consisted of 125 mM NaCl, 0.5 mM MgCl_2_, 4.5 mM KCl, 10 mM NaHCO_3_, 2 mM CaCl_2_, 5 mM glucose, 20 mM sucrose, and 10 mM HEPES, pH 7.4, 300 mOsml/kg. To make hypotonic or hypertonic AAH, the NaCl concentration was either decreased or increased, respectively, to achieve a final osmolarity of 250 or 350 mOsml/kg.

### 4.2. Lens Dissection

Here, 6–8 week-old C57BL/6 mice were supplied by the Vernon Jansen Unit located in the Faculty of Medical and Health Sciences at the University of Auckland or purchased from Sankyo Laboratory Service Corporation (Tokyo, Japan). Mice were housed under temperature-controlled conditions (23 ± 5 °C), under a 12 h light (8:00–20:00) and dark (20:00–8:00) cycle. All animals in this study were handled in accordance with the Association for Research in Vision and Ophthalmology (ARVO) Statement for the Use of Animals in Ophthalmic and Vision Research, and all procedures were approved by either the University of Auckland Animal Ethics Committee (AEC#001893) or the Keio University Animal Research Committee (#11014-5). Mice were euthanized with 5% isoflurane by inhalation (Wako Pure Chemical Industries Ltd., Osaka, Japan), the globe of the eye removed and then dissected in one of two ways. To prepare lenses for in vitro culture, the enucleated eyes were dissected from the posterior pole by cutting four flaps starting from the optic nerve head and extending the cuts toward the limbus. The four flaps were removed by cutting at the lens–zonules intersection and removing all adherent tissues and zonules. To prepare lenses for in situ culture with their zonules intact, a small hole (2 mm diameter) was first made in the cornea of enucleated eyes to allow the access of pharmacological reagents and at the end of the experiment, fixative. After both dissection protocols, lenses were either immediately fixed or organ-cultured in AAH in the absence or presence of pharmacological reagents for up to 120 min.

### 4.3. Lens Organ Culture

Lenses prepared in vitro or in situ were both organ-cultured in a humidified atmosphere containing 5% CO_2_ (HERA incubator, ESPEC Corporation, Osaka, Japan) at 37 °C for up to 120 min using four time points in Isotonic AAH in the absence or presence of a variety of pharmacological reagents and for 120 min in hypotonic and hypertonic AAH. Zonular tension was modulated pharmacologically in lenses prepared in situ by the application of the muscarinic receptor antagonist tropicamide, which causes the relaxation of the muscles of the ciliary body and therefore increases zonular tension, or the agonist pilocarpine, which by inducing contraction of the ciliary muscle moves the ciliary body forward to reduce the tension applied to the lens by the zonules. Tropicamide and pilocarpine were used at 1:5 dilution prepared in an AAH buffer from 1% *w*/*v* eye drops, and the lenses were incubated for 120 min. To examine the effects of TRPV1 and TRPV4 activators on the subcellular localization of TRPV1/4 receptors, lenses prepared in situ were organ-cultured in the presence of 10 μM Capsaicin or 1 μM GSK101 and incubated for 45 min.

### 4.4. Immunohistochemistry

Lenses were fixed in 0.75% paraformaldehyde (PFA) prepared in PBS pH 7.4 at room temperature for 24 h. After fixation, PFA was removed by washing in PBS pH 7.4. The lenses were then prepared for cryosectioning by consecutive 1 h incubations in 10% and then 20% sucrose prepared in PBS pH 7.4 at room temperature. Cryoprotected lenses were left overnight for up to a maximum of 1 week in 30% sucrose at 4 °C, before cutting 14 μM thick axial cryosections using a Cryostat (CM1950 Leica Biosystems, Wetzlar, Germany). Cryosections were incubated in blocking solution (3% bovine and 3% goat serum) for 1 h at room temperature and then incubated overnight at 4 °C in primary antibodies specific for either TRPV1 or TRPV4, which were prepared in blocking solution. Following washing in PBS pH 7.4, sections were then incubated goat antirabbit Alexa Fluor 488 secondary antibody prepared in blocking buffer, which contained 0.125 μg/mL of the nuclear marker DAPI for 1 h at room temperature. Sections were again washed in PBS pH7.4 and incubated with WGA Alexa 594 for 1 h at room temperature to label the cell membranes. Following a final wash in PBS pH 7.4, sections were mounted with a cover slip using VectaShield HardSet Antifade Mounting Medium (Vector Laboratories, Burlingame, CA, USA) and imaged using laser scanning confocal microscopes equipped with FluoView software (Olympus FV-1000 or FV-3000, Tokyo, Japan). The images presented for each experimental protocol are representative of sections taken from a minimum of three lenses.

## 5. Conclusions

In conclusion, we have shown the trafficking of the mechano-sensitive channels TRPV1 and TRPV4 can be dynamically regulated by alterations in zonular tension, exposure to osmotic challenge and the application of pharmacological activators. Since the activation of TRPV1 and TRPV4 has been implicated in the regulation of lens water transport [7,11] and lens water transport has emerged as a critical parameter that can modulate the transparency and refractive properties of the lens [2], further studies into how TRPV1/4 membrane trafficking is regulated have the potential to improve our understanding into how the optical properties of the lens are actively maintained as it grows throughout life and how they are impaired during the development of lens cataract.

## Data Availability

Upon request, we will make the data available to other researchers.
